# Einsatz von Albumin: State of the art

**DOI:** 10.1007/s00063-021-00875-4

**Published:** 2021-10-07

**Authors:** Timo Mayerhöfer, Christian J. Wiedermann, Michael Joannidis

**Affiliations:** 1 Gemeinsame Einrichtung für Intensiv- und Notfallmedizin, Abteilung für Innere Medizin I, Medizinische Universität Innsbruck, Anichstraße 35, 6020 Innsbruck, Österreich; 2 Institut für Public Health, Medical Decision Making und HTA, UMIT Private Universität für Gesundheitswissenschaften, Medizinische Informatik und Technik, Hall in Tirol, Österreich; 3 Institut für Allgemeinmedizin, Landesfachhochschule für Gesundheitsberufe Claudiana, Bozen, Italien

**Keywords:** Volumentherapie, Sepsis, Aszites, Leberzirrhose, Hämodynamik, Fluid therapy, Sepsis, Ascites, Liver cirrhosis, Hemodynamics

## Abstract

Die Saline vs. Albumin Fluid Evaluation (SAFE) Studie hat gezeigt, dass der Einsatz von Albumin als Infusionslösung im Bereich der Volumentherapie fast überall sicher ist. Eine Ausnahme stellt hypoonkotisches Albumin beim Schädel-Hirn-Trauma dar. Während für Patientinnen und Patienten mit Leberzirrhose klare Indikationen existieren, fehlen für andere Einsatzgebiete noch große Studien, die einen klinisch relevanten Vorteil jenseits der hämodynamischen Wirksamkeit zweifelsfrei zeigen konnten und damit einen breiteren Einsatz rechtfertigen würden. Bei der „Large-volume“-Parazentese, der spontanen bakteriellen Peritonitis, aber auch beim hepatorenalen Syndrom ist der Einsatz von Albumin aufgrund eines klinischen Benefits in randomisierten kontrollierten Studien klar empfohlen und etabliert. Beim septischen Schock kann ein Einsatz von Albumin in Erwägung gezogen werden, wobei sich zwei große Studien zu dieser Fragestellung in Deutschland und Italien noch in der Rekrutierungsphase befinden. Für viele Einsatzgebiete in der Volumentherapie gilt, dass Albumin vor allem dann eingesetzt werden kann, wenn andere Maßnahmen zur hämodynamischen Stabilisierung bereits ausgeschöpft sind. Das gilt sowohl für die Volumengabe bei Hypovolämie als auch für das konservative Volumenmanagement einschließlich der sog. Deresuscitation-Phase. Inwieweit die Korrektur einer ausgeprägten Hypoalbuminämie durch Gabe von exogenem Albumin auch das schlechtere Outcome solcher Patientinnen und Patienten verbessert, ist ebenfalls Teil laufender Studien. Auf dem Weg zu einem Mehr an individualisierter Therapie kann in Zukunft die Hypoalbuminämie bei Entscheidungen für oder gegen einen Einsatz von intravenösen Albuminlösungen in der Volumentherapie eine wichtige Rolle einnehmen.

## Abstract (en)

The Saline versus Albumin Fluid Evaluation (SAFE) study has demonstrated that the use of albumin as an infusion solution in volume therapy can be regarded as safe. An exception is hypo-oncotic albumin in traumatic brain injury. While clear indications of albumin exist for some patients with liver cirrhosis, large studies that demonstrate a clinically relevant advantage beyond hemodynamic effects and would therefore justify wider use in many other areas are still lacking. In large-volume paracentesis, spontaneous bacterial peritonitis, but also in hepatorenal syndrome, use of albumin is recommended and established due to clinical benefit in randomized controlled trials. In septic shock, use of albumin may be considered, with two large studies addressing this issue in Germany and Italy being still in the recruitment phase. For volume therapy, albumin can be used primarily when other measures for hemodynamic stabilization have been exhausted. This applies to volume resuscitation in hypovolemia as well as in conservative fluid management in the so-called “de-resuscitation” phase. The extent to which the correction of severe hypoalbuminemia with exogenous albumin can improve the impaired outcome of these patients is also part of ongoing studies. On the way to a more individualized medicine, hypoalbuminemia may serve as a parameter in future decision making for or against the use of albumin in volume therapy.

## Hintergrund

Nach Bekanntwerden wissenschaftlichen Fehlverhaltens eines deutschen Forschers im Jahr 2010 [65], der schwerpunktmäßig die Anwendung von Hydroxyethylstärke (HES) untersuchte, und rezenten Warnhinweisen von der European Medicines Agency (EMA) und US Food and Drug Administration (FDA) [19, 21], gerät Humanalbumin (HA) als Alternative für Kolloide in der Volumentherapie immer mehr in den Fokus. Während für viele Einsatzgebiete klare und durch Studien belegte Empfehlungen existieren, stellt sich die Studienlage in anderen Bereichen als eher heterogen dar, wobei viele Fragen noch nicht abschließend geklärt sind. HA wird zur hämodynamischen Stabilisierung verabreicht oder wenn Evidenz für eine Reduktion der Gesamtmortalität im Indikationsgebiet aus randomisierten kontrollierten Studien abgeleitet werden kann. In diesem Artikel sollen die physiologischen Funktionen von Albumin und verschiedene denkbare Einsatzgebiete von HA, vor allem im Bereich der Volumentherapie, diskutiert werden.

### Biochemie und Physiologie von Albumin

**Figure 1:**
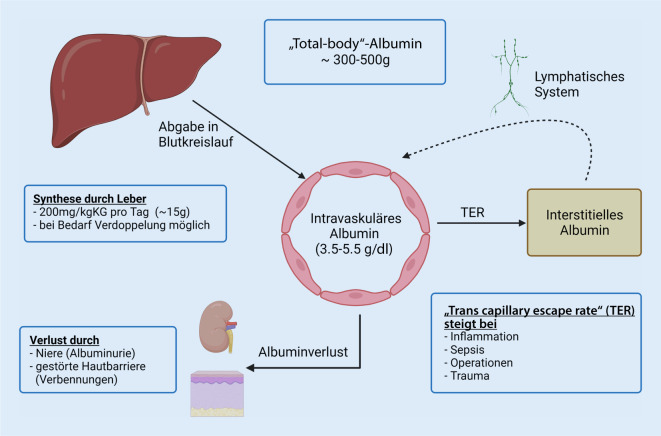

Albumin ist ein komplexes Protein mit zahlreichen verschiedenen Funktionen im menschlichen Körper. Eine der wichtigsten Aufgaben von Albumin ist die Aufrechterhaltung des onkotischen Drucks [13]. Zudem werden ihm immunmodulatorische und antioxidative Eigenschaften zugeschrieben [41]. Eine wichtige Rolle spielt Albumin außerdem als Transportprotein. Albumin besteht aus etwa 600 Aminosäuren und liegt in einer flexiblen Struktur vor, die verschiedenste Bindungen ermöglicht. Ungefähr 10–15 g Albumin werden pro Tag von der Leber synthetisiert und nach intravaskulär abgegeben (Abb. 1), wo Albumin den größten Anteil an Plasmaproteinen stellt. Ein Teil davon wird durch Kapillaren in das Interstitium abgegeben und gelangt über die Lymphbahnen wieder zurück in den Blutkreislauf. Dieser Verlust von onkotisch wirksamem Plasmaalbumin durch die Kapillaren ins Interstitium, der einen höheren extravaskulären Albumingehalt zur Folge hat, kann sich bei systemischer Inflammation, unter anderem bei Sepsis, nach Operationen oder traumatischen Ereignissen, entsprechend vergrößern [23].

### Allgemeines und Sicherheit von Albumin

Aufgrund des hohen Preises und fehlender Daten, die einen generellen klinischen Vorteil gegenüber Kristalloiden belegen würden, gibt es für Intensivpatientinnen und -patienten für die Gabe von HA keine allgemeine Empfehlung in der Volumentherapie [20]. Eine wichtige nach dem Auftauchen von Risikosignalen aus Metaanalysen zu klärende Frage, war jedoch die generelle Sicherheit von HA als Volumentherapeutikum [15]. Daher wurde im Jahr 2004 die „Saline vs Albumin Fluid Evaluation“ (SAFE)-Studie durchgeführt, die 6997 kritisch kranke Menschen generell mit hypoonkotischem HA (4 %) oder mit gewöhnlicher Kochsalzlösung behandelte [50]. Der Volumeneffekt von HA war besser als jener von Kristalloiden. So lag das Verhältnis verabreichter Volumina von HA zu Kristalloiden, die in der SAFE-Studie bei Intensivpatientinnen und -patienten zum Erreichen hämodynamischer Endpunkte über die ersten 4 Tage benötigt wurden, bei 1:1,4 [50]. Dieses Verhältnis zu Kristalloiden scheint besser als jenes von HES zu sein, das nach großen Studien mit 1:1,2 bis 1:1,1 angegeben wird [46]. In der SAFE-Studie konnten zwar keine Mortalitätsunterschiede zwischen den beiden Gruppen gezeigt werden, jedoch schien die Verabreichung von HA für die meisten Patientinnen- und Patientengruppen sicher zu sein. Einschränkend war bei Traumapatientinnen und -patienten mit hypoonkotischem HA ein Trend zu höherer Mortalität erkennbar, der durch eine Post-hoc-Analyse für das Schädel-Hirn-Trauma (SHT) bestätigt wurde. Besonders HA 4 % sollte daher bei diesen Personen vermieden werden [51]. Unterschiedliche Empfehlungen gibt es für hyperonkotisches (20–25 %) HA beim SHT. Die European Society of Intensive Care Medicine (ESICM) rät generell von einer Verwendung von HA in der Volumentherapie bei Neurointensivpatientinnen und -patienten ab. In skandinavischen Leitlinien wird im Rahmen des sog. Lund-Konzepts für die Therapie von Patientinnen und Patienten mit schwerem SHT weiterhin HA 20–25 % empfohlen [44].

Konzeptuell sind beim hyperonkotischen HA stärkere Volumeneffekte zu erwarten. Zur Klärung dieser Annahme verglich die SWIPE-Studie, die hämodynamisch instabile Patientinnen und Patienten einschloss, HA unterschiedlicher Konzentrationen. Hierbei zeigte sich in Bezug auf das verabreichte Volumen ein Vorteil des HA 20 % gegenüber HA 4–5 % und eine niedrigere kumulative Flüssigkeitsbilanz ohne eine höhere Nebenwirkungsrate. Personen mit SHT waren hier jedoch ausgeschlossen [38].

Einen häufig diskutierten Aspekt bei der Verabreichung von HA stellt die Natriumbelastung dar. Es existieren unterschiedliche Lösungen, für die der Natriumgehalt teilweise nur als Bereich angegeben ist (siehe Tab. 1). Bei HA 20 % reicht dann der Natriumgehalt z. B. von 100–130 mmol/l. Bei einem verabreichten Volumen von 300 ml/d entspricht dies einer Natriumbelastung von 30–39 mmol pro Tag.

**Table 1:** 

Infusionslösung	Konzentration (g/l)	Natriumgehalt (mmol/l)	Hersteller	Land
Human Albumin Baxter 250/200/50 g/l Infusionslösung	250/200/50	130–160/100–130/130–160	Baxter AG, Wien, Österreich/TAKEDA GmbH, Konstanz, Deutschland	A/D
Human Albumin CSL Behring 20 % Infusionslösung	200	125	CSL Behring GmbH, Hattersheim, Deutschland	A/D
Alburex 20/5	200/50	140	CSL Behring GmbH, Hattersheim, Deutschland	A/D
Crealb 200/40 g/l	200/40	100	Sanquin Plasma Products, Amsterdam, Niederlande	A/D
Flexbumin 250/200 g/l Infusionslösung	250/200	130–160	Baxter AG, Wien, Österreich/Shire Deutschland GmbH, Berlin, Deutschland	A/D
Albiomin 200/50 g/l Infusionslösung	200/50	122/145	Biotest Pharma GmbH, Dreieich, Deutschland	A
Albunorm 250/200/50/40 g/l Infusionslösung	250/200/50/40	144–160	Octapharma Pharmazeutika Produktionsges.m.b.H, Langenfeld, Deutschland	A
Human Albumin Immuno 20 % Infusionslösung	200	100–130	Baxter AG, Wien, Österreich	A
Human Albumin Octapharma 25 % Infusionsflasche	250	142–158	Octapharma Pharmazeutika Produktionsges.m.b.H, Wien, Österreich	A
Plasma Protein Lösung 5 % Infusionsflasche	50	142–158	Octapharma Pharmazeutika Produktionsges.m.b.H, Wien, Österreich	A
Albiomin 20 %/5 %	200/50	–	Biotest Pharma GmbH, Dreieich, Deutschland	D
Albunorm 25/20/5/4 %	250/200/5/4	144–160	Octapharma GmbH, Langenfeld, Deutschland/Orifarm GmbH, Leverkusen, Deutschland	D
Albutein 200/50 g/l	200/50	145	Grifols Deutschland GmbH, Frankfurt am Main, Deutschland	D
Human Albumin 250/200/50 g/l Baxalta	250/200/50	130–160/100–130/130–160	TAKEDA GmbH Konstanz, Deutschland/Shire Deutschland GmbH, Berlin, Deutschland	D
Humanalbin	50	155	CSL Behring GmbH, Hattersheim, Deutschland	D
Humanalbumin 200g/l Kedrion	200	123,5–136,5	Kedrion Biopharma GmbH, Gräfelfing, Deutschland	D
Plasbumin 25/20	250/200	145	Grifols Deutschland GmbH, Frankfurt am Main, Deutschland	D

*A* Österreich, *D* Deutschland

^a^Bundesamt für Sicherheit im Gesundheitswesen (23.07.2021). Verfügbar unter: https://aspregister.basg.gv.at/aspregister/faces/aspregister.jspx;jsessionid=UwLNZCUUr4OBedVO_a4CxHCQA7clzjKlivdVF_sGTElRJrfAq8UT!-1073206191; Paul-Ehrlich-Institut (23.07.2021). Verfügbar unter: https://www.pei.de/DE/arzneimittel/blutprodukte/albumine/albumine-node.html. Namensgleiche bzw. ähnliche Humanalbuminlösungen sind gebündelt dargestellt, verschiedene verfügbare Konzentrationen (und der dazugehörige Natriumgehalt) sind durch Schrägstriche voneinander getrennt

#### Merke.

Im Vergleich zu künstlichen Kolloiden erhöht hyperonkotisches Albumin die Volumenwirksamkeit ohne zusätzliche Nephrotoxizität.

#### Merke.

Albumin 4–5 % sollte beim Schädel-Hirn-Trauma nicht verwendet werden.

### Hypoalbuminämie

Die Hypoalbuminämie (Serumalbumin < 3,5 g/dl) ist ein häufiges Phänomen bei kritisch kranken Patientinnen und Patienten, vor allem bei Infektionen, Tumorerkrankungen, aber auch bei großen Operationen oder schweren traumatischen Verletzungen und ist mit einer schlechten Prognose assoziiert [62]. Dies gilt, wie rezent gezeigt werden konnte, auch für an „coronavirus disease 2019“ (COVID-19) Erkrankte [6]. Der Albuminspiegel scheint außerdem einen Einfluss auf die Pharmakokinetik und Pharmakodynamik verschiedener Antibiotika zu haben [62]. Ob eine Albuminsubstitution bei Hypoalbuminämie generell Sinn macht, wurde in einzelnen Pilotstudien untersucht und zeigte sich hier als vorteilhaft [17]. Eine Subgruppenanalyse der bereits erwähnten SAFE-Studie, in der zirkulierende Albuminspiegel in der Albumingruppe durchaus höher waren als in der Kontrollgruppe, konnte solche Vorteile nicht zeigen und stellte unabhängig vom Baseline-Albuminwert keine Unterschiede fest. Allerdings ist die Hypoalbuminämie in der Studie durch die verabreichte Albuminmenge nicht gänzlich korrigiert worden: so lag der mittlere Albuminspiegel am Tag 4 mit 2,9 g/dl im Vergleich zu 2,3 g/dl bei der Kontrollgruppe immer noch im hypoalbuminämischen Bereich [22]. Ebenfalls keinen Mortalitätsvorteil zeigte die randomisierte, offene „Volume-replacement-with-albumin-in-severe-sepsis“(ALBIOS)-Studie 9, die der SAFE-Studie folgte. Für Patientinnen und Patienten im Albuminarm war hier als Zielparameter eine Serumalbuminkonzentration von 3 g/dl definiert. Es wurden, im Gegensatz zur SAFE-Studie, jedoch HA 20 % verwendet und nur Personen mit schwerer Sepsis (s. im Folgenden) eingeschlossen.

#### Merke.

Ohne zusätzliche Indikation sollte Albumin bei Intensivpatientinnen und -patienten (derzeit) nicht allein zum Ausgleich einer Hypoalbuminämie verwendet werden.

### Albumin bei der Sepsis

Die SAFE-Studie lieferte Hinweise, dass die Subgruppe der Personen mit schwerer Sepsis von einer HA-Gabe profitieren könnte. Daher untersuchte man in der erwähnten ALBIOS-Studie nur solche mit schwerer Sepsis oder septischem Schock und behandelte diese entweder mit HA 20 % oder nur mit Kristalloiden. Wiederum zeigte sich kein eindeutiger Unterschied in der Mortalität, jedoch gab es in einer Post-hoc-Analyse Hinweise auf einen Vorteil für Patientinnen und Patienten unter Albumintherapie im septischen Schock [9]. Die Ergebnisse der „Early-albumin-resuscitation-in-septic-shock“(EARSS)-Studie, die nur Personen im septischen Schock einschloss, fand laut ersten Berichten eine etwas bessere Mortalität, wegen einer zu geringen Zahl von eingeschlossenen Studienteilnehmern ohne statistisch signifikanten Unterschied. Die Ergebnisse sind jedoch nie vollständig publiziert worden [12]. Eine gepoolte Analyse dieser 3 großen Studien (ALBIOS, EARSS, SAFE) errechnete einen Vorteil für Patientinnen und Patienten mit schwerer Sepsis unter HA [64]. In Deutschland schließt nun die „Albumin-replacement-in septic-shock“(ARISS)-Studie Personen im septischen Schock ein, befindet sich allerdings noch in der Rekrutierungsphase [52].

In den Leitlinien der Surviving Sepsis Campaign beschränkt sich die Empfehlung zum Einsatz von HA somit auf Patientinnen und Patienten mit schwerer Sepsis oder septischem Schock, die eine große Menge an Volumen benötigen [48]. Für COVID-19-Erkrankte im Schock gibt es ebenfalls keine Empfehlung zur Routinegabe von HA; von der Verwendung künstlicher Kolloide wird abgeraten [1].

Primär sollten bei Sepsis auf der Intensivstation also weiterhin Kristalloide verwendet werden [48]. Ist ein adäquater Volumenstatus dadurch nicht zu erreichen und werden erhebliche Mengen an Kristalloiden benötigt, kann HA zusätzlich verabreicht werden [39, 48]. Humanalbumin wird bei dieser Indikation also nicht routinemäßig empfohlen, da ein Überlebensvorteil, bisher nicht einhellig gezeigt werden konnte [9]. Laufende Studien sollten hier in Zukunft Antworten liefern.

#### Merke.

Ist im Rahmen einer Sepsis mit Kristalloiden kein ausreichender Volumenstatus zu erreichen, kann der Einsatz von Albumin erwogen werden.

### Albumin bei der Leberzirrhose

Bei den unterschiedlichen Einsatzgebieten von HA im Rahmen der Leberzirrhose kann zwischen HA als Medikament und HA als Volumenersatz unterschieden werden. Empfehlungen gibt es vor allem nach einer „Large-volume“-Parazentese im Rahmen eines Aszites, bei der spontan-bakteriellen Peritonitis (SBP) sowie beim hepatorenalen Syndrom (HRS).

### *Large-volume-Parazentese*

Eine klare Empfehlung zur Gabe von HA gibt es im Rahmen eines Aszites, der durch eine Zirrhose verursacht wurde und rasch punktiert werden muss. Um hämodynamische Probleme zu vermeiden, ist eine HA-Gabe vor allem dann wichtig, wenn die Parazentese eine Menge von 5 l überschreitet [47]. Dann sollte HA in einer Dosis von 8 g/l entfernten Aszites infundiert werden [24, 45].

Bei Patientinnen und Patienten mit instabilem Kreislauf oder mit akuter Nierenschädigung sollte die therapeutische Parazentese auch dann mit einer HA-Infusion verbunden werden, wenn das Aszitesvolumen geringer als 5 l ist, da für die Nierenfunktion eine stabile Hämodynamik besonders wichtig ist und HA hier zu einer Verbesserung von Nierenfunktion und Mortalität führen kann [7, 35].

Das Wiederholen der „Large-volume“-Parazentese gilt außerdem als wirksame Methode zur Behandlung von refraktärem Aszites und wird hier als Erstlinientherapie empfohlen [10, 35]. In diesem Rahmen kann außerdem eine Langzeitgabe von HA in Erwägung gezogen werden, die in einer nichtrandomisierten Studie einen Vorteil gezeigt hat [16].

Eine weitere Empfehlung für die HA-Gabe, die jedoch nicht zur Volumentherapie gezählt werden kann, gibt es bei schweren Muskelkrämpfen, wie sie bei Zirrhosepatientinnen und -patienten häufig vorkommen. Hier kann HA, neben Baclofen, in einer Dosierung von 20 g/Woche eingesetzt werden [35].

### *Langzeitgabe von Albumin*

Die Langzeitgabe von HA bei Leberzirrhose und unkompliziertem Aszites ist eher als medikamentöse Therapie und nicht als Volumentherapie im eigentlichen Sinn zu werten. Untersuchungen hierzu haben zu unterschiedlichen Studienergebnissen geführt. In der „Human-albumin-for-the-treatment-of-ascites-in-patients-with-hepatic-cirrhosis“(ANSWER)-Studie führte eine wöchentliche HA-Gabe zur Reduktion von Mortalität und Komplikationen [11]. Eine allgemeine Empfehlung für die Langzeitgabe von HA existiert im Moment aber noch nicht [8]. In 2 anderen Studien, „midodrine and albumin for prevention of complications in patients with cirrhosis awaiting liver transplantation“ (MACHT) und „a randomized trial of albumin infusions in hospitalized patients with cirrhosis“ (ATTIRE), die jedoch in Bezug auf Studiendesign und HA-Gabe nur bedingt vergleichbar sind, konnte ein Vorteil wie in ANSWER nicht nachgewiesen werden [14, 54].

Eine Langzeit-HA-Therapie kommt somit als Behandlungsoption bei Patientinnen und Patienten mit Aszites von mindestens Grad 2 (moderat) unter Umständen dann infrage, wenn diese auf moderate Dosen von Diuretika (mindestens 200 mg/Tag eines Aldosteronantagonisten und 25 mg/Tag Furosemid) nicht ausreichend ansprechen [11].

### *Spontan-bakterielle Peritonitis*

Die SBP ist als Aszitesinfektion ohne chirurgisch behandelbare Quelle definiert [56]. Neben der empirischen Antibiotikatherapie können Patientinnen und Patienten mit Aszites und SBP von einer Behandlung mit HA profitieren. In einer älteren Studie konnten für diese die Notwendigkeit von Nierenersatzverfahren verringert und die Mortalität gesenkt werden [55]. Besonders scheinen Personen mit SBP und Nierenfunktionsstörung (Serumkreatinin > 1 mg/dl) oder hohen Bilirubinwerten (Gesamtbilirubin > 4 mg/dl) zu profitieren. Die Behandlung wurde mit hypertonem HA in einer Dosierung von 1,5 g/kgKG innerhalb von 6 h nach der Diagnose gefolgt von 1 g/kgKG am 3. Tag nach Diagnose durchgeführt [10, 20, 24, 45].

Im Gegensatz dazu hat eine Metaanalyse keinen Vorteil bei Zirrhosepatientinnen und -patienten mit extraperitonealen Infektionen zeigen können [32].

### *Hepatorenales Syndrom*

Beim HRS kommt es bei Personen mit akuter oder chronischer Lebererkrankung zur akuten Nierenschädigung. Das HRS stellt eine Ausschlussdiagnose dar und muss von anderen Ursachen für eine akute Nierenschädigung (AKI), wie Hypovolämie oder nephrotoxische Medikamente, als Verursacher bei Personen mit Lebererkrankung abgegrenzt werden [3].

Beim HRS selbst wird zwischen HRS Typ 1 (HRS-AKI) und HRS Typ 2 (HRS-NAKI) unterschieden, wobei die HRS-AKI die schwerere Verlaufsform darstellt und durch Kreatininwerte über 2,5 mg/dl definiert ist.

Die Behandlung mit HA und einem Vasopressor ist beim HRS-AKI indiziert und wirksam. Empfohlen wird HA am Tag 1 in einer Dosierung von 1 g/kgKG, gefolgt von 20–40 g täglich über 2–16 Tage [24].

Terlipressin plus HA gilt als Therapie der ersten Wahl zur Behandlung von HRS-AKI. Auch eine Gabe von Noradrenalin wäre denkbar und vor allem aufgrund des günstigeren Preises interessant. Jedoch haben Arora et al. [4] für Terlipressin einen Überlebensvorteil gegenüber Noradrenalin bei Patientinnen und Patienten mit HRS-AKI zeigen können.

Terlipressin (1 mg alle 4–6 h) in Kombination mit HA (20–40 g/Tag) wird nicht nur zur Behandlung des HRS-AKI, sondern auch von HRS-NAKI empfohlen und scheint auch bei geringer ausgeprägtem Schweregrad der Nierenschädigung einen Vorteil zu bringen [20, 53].

#### Merke.

Beim HRS-AKI gilt Albumin in Kombination mit Terlipressin als Standardtherapie.

### Albumin bei der akuten Nierenschädigung

Auf der Basis jüngst aktualisierter behördlicher Bewertungen von EMA und FDA muss HES unabhängig von Molekülgroße und Substitutionsgrad mittlerweile als nephrotoxisch angesehen werden [19, 21]. Der pathophysiologische Mechanismus (u. a. osmotische Nephrose) könnte potenziell auch bei anderen Kolloiden zu Tragen kommen. Bei Albumin hingegen gilt, dass es selbst in großen Studien keine Risikosignale in diese Richtung gab und HA daher in Bezug auf Nephrotoxizität als sicher angesehen werden kann [9, 50]. Nachdem durch eine Metaanalyse gezeigt werden konnte, dass die Hypoalbuminämie mit einem erhöhten Risiko einer akuten Nierenschädigung assoziiert ist [26], könnte sich die Gabe von HA unter Umständen bei diesen Patientinnen und Patienten im Sinne einer Prävention als vorteilhaft zeigen. Klare Empfehlungen gibt es für das HRS-AKI [24], bei SBP und, im Bereich der Herzchirurgie, erste Daten für die Prophylaxe der AKI [33]. Da Albumin als Transportprotein für Furosemid die Verfügbarkeit in der Niere erhöht, kann es bei Hypoalbuminämie die Wirksamkeit von Schleifendiuretika verbessern. Dementsprechend konnten in einer kleinen randomisierten Studie durch HA in Kombination mit Furosemid eine verbesserte negative Flüssigkeitsbilanz und eine Verbesserung der Oxygenierung beim Acute Respiratory Distress Syndrome (ARDS) erreicht werden [40]. Eine Metaanalyse kommt jedoch zu dem Schluss, dass es sich bei der Überwindung der Diuretikaresistenz hypoalbuminämischer Personen durch HA-Gabe nur um einen transienten Effekt handle [30]. Ob Albumin im Bereich der Nephroprotektion einen generellen Nutzen mit sich bringt, muss noch durch große Studien untersucht werden.

### Albumin bei Nierenersatztherapie

Neue Studiendaten zum Einsatz von HA in der Nierenersatztherapie existieren für die Behandlung der intradialytischen Hypotonie (IDH; [36]) und für die Verbesserung des Flüssigkeitsentzugs unter kontinuierlicher Nierenersatztherapie (CRRT; [43]).

Eine im Jahr 2021 veröffentliche randomisierte „Cross-over“-Studie liefert Hinweise, dass hospitalisierte Personen mit Hypoalbuminämie von der Gabe von HA 20–25 % profitieren könnten. In der Studie wurde jeweils zu Beginn der Dialyse randomisiert und es wurden 100 ml von entweder 0,9 % NaCl oder HA 25 % intravenös verabreicht. Die HA-Gabe vor der Dialyse führte zu weniger Episoden von Hypotonie [36].

Außerdem kam eine kürzlich publizierte Sekundäranalyse der RENAL-Studie zu dem Schluss, dass unter CRRT mittels Gabe von HA 20 % bei hypoalbuminämischen Patientinnen und Patienten eine stärkere negative Flüssigkeitsbilanz zu erzielen war und die HA-Gabe in diesem Einsatzgebiet als sicher angesehen werden kann [43]. Insgesamt fehlen für die unterschiedlichen Behandlungsziele des intravenösen Einsatzes von HA während der Nierenersatztherapie noch große randomisierte kontrollierte Studien [27].

### Albumin beim ARDS

Beim ARDS haben sich niedrige Albuminspiegel ebenfalls als schlechter prognostischer Marker erwiesen und sind mit der Entwicklung von Ödemen assoziiert [28]. Restriktive Strategien in der Volumentherapie mit Flüssigkeitsbeschränkung unter Überwachung des extravaskulären Lungenwassers, des pulmonalkapillaren Verschlussdrucks oder des zentralen Venendrucks und eine auf die Diurese abgestimmte Furosemidgabe inklusive HA-Gabe bei hypoproteinämischen Patientinnen und Patienten verbesserten beim ARDS die Oxygenierung signifikant und verkürzten die Dauer der mechanischen Beatmung, hatten jedoch keinen signifikanten Einfluss auf die Mortalität [58]. In einer randomisierten kontrollierten Studie zur HA-Gabe bei ARDS, in der die Kombination aus Furosemid und HA mit alleinigem Furosemid verglichen wurde, zeigte sich ein Vorteil in Bezug auf eine bessere Oxygenierung und eine größere negative Flüssigkeitsbilanz über die Studiendauer (7 Tage), wobei hier einschränkend erwähnt werden muss, dass lediglich 40 Patientinnen und Patienten mit ARDS eingeschlossenen wurden [29, 40]. Eine Metaanalyse bestehend aus 3 randomisierten kontrollierten Studien kam im Jahr 2014 zu dem Schluss, dass HA im Vergleich zu Kristalloiden zwar die Oxygenierung, jedoch nicht die Mortalität verbessert und dass hier dringend größere Studien nötig sind, um den beschriebenen Benefit zu belegen [57].

### Albumin in der Chirurgie

Rezente Tracer-Studien zeigten, dass es im Rahmen von großen abdominellen Eingriffen innerhalb der ersten Stunde nach Beginn der Operation zu transkapillaren Albuminverlusten kommt. Dementsprechend kann perioperativ ein Abfall des Albuminspiegels im Serum beobachtet werden [2, 31]. Eine präoperative Hypoalbuminämie kann dadurch postoperativ signifikant zunehmen. Für Erwachsene im perioperativen Setting gilt, dass HA nicht generell bei Hypovolämie oder zur hämodynamischen Stabilisierung empfohlen ist, zumal ein allgemeiner Vorteil im Vergleich zu Kristalloiden nicht gezeigt werden konnte. Sollten andere therapeutische Maßnahmen jedoch bereits ausgeschöpft sein, kann HA in Betracht gezogen werden [20].

Nach einer älteren Empfehlung, die nicht aktualisiert wurde, kann HA als postoperativer Volumenexpander bei Menschen verwendet werden, die sich einer größeren Operation unterzogen haben und bei denen nach Normalisierung des Kreislaufvolumens das Serumalbumin < 2 g/dl bleibt [34].

In der Herzchirurgie kann HA 5 % zur Korrektur einer Hypovolämie, zur hämodynamischen Stabilisierung sowie zum sog. Priming (Vorfüllen) der Herz-Lungen-Maschine verwendet werden [20, 49]. Beim Priming zeigte sich für HA gegenüber Kristalloiden ein Vorteil, da unter Kristalloiden das Serumlaktat anstieg und höhere Flüssigkeitsmengen intraoperativ nötig gewesen sind als im Vergleich mit HA 5 % [60]. Zudem zeigten sich in einer Studie von Lee et al. [33] Vorteile von HA in Bezug auf die postoperative AKI-Rate. Personen mit einem Serumalbumin von unter 4 g/dl wurde dabei eine an die jeweilige Albuminkonzentration angepasste HA-Dosis verabreicht. Die Gabe führte zu einer höheren Urinausscheidung während und zu einer niedrigeren AKI-Rate nach der Operation.

Studiendaten, die einen Vorteil von HA gegenüber Kristalloiden nahelegen, gibt es außerdem für Patientinnen und Patienten unter venoarteriellen extrakorporalen Membranoxygenierung (ECMO) und HA könnte hier zu einem besseren Überleben beitragen. Es handelt sich jedoch um eine retrospektive Auswertung, die noch durch weitere Studien bestätigt werden muss [61].

### Albumin bei Verbrennungen

Schwere Verbrennungen sind durch einen starken Proteinverlust gekennzeichnet [59], weshalb mehrere Studien die Verwendung von HA in diesem Bereich untersuchten. Wie bei anderen Einsatzgebieten für HA kommen diese jedoch zu verschiedenen Ergebnissen. Metaanalysen haben einen geringen [42] oder keinen Effekt [18] der HA-Gabe auf die Mortalität gezeigt und hatten dabei teils Studien unterschiedlicher Qualität eingeschlossen. Bei Verbrennungspatientinnen und -patienten wird daher die Gabe von HA zur hämodynamischen Stabilisierung nicht primär empfohlen, kann aber, wenn große Mengen an Kristalloiden benötigt werden, in Erwägung gezogen werden [20]. Als Zielgröße kann hier eine Serumalbuminmindestkonzentration von 2,5 g/dl dienen, wie sie in der rezent aktualisierten S2k-Leitlinie der Deutschen Gesellschaft für Verbrennungsmedizin vorgeschlagen wird [5].

Die „International Fluid Academy“ (IFA) empfiehlt HA 20 % bei schweren Verbrennungen, vor allem in der sog. Deresuscitation-Phase [63], also nach initialer Stabilisierung, um dem „fluid creep“ entgegen zu wirken [37].

### Weitere Indikationen

Wie bereits erwähnt wird HA 20 % im Rahmen des SHT als Teil des sog. Lund-Konzepts im skandinavischen Raum eingesetzt [25], von der European Society of Intensive Care Medicine (ESICM; [44]) aber weder in 5 %iger noch in 20 %iger Konzentration empfohlen.

Die Gabe von HA kann laut einer italienischen Leitlinie außerdem unter folgenden Umständen bei Menschen mit Durchfall sinnvoll sein, die keine enterale Ernährung vertragen: Durchfallvolumen > 2 l, ein Serumalbumin < 2 g/dl und, trotz Gabe von kurzkettigen Peptiden und Mineralstoffpräparaten, anhaltende Durchfallsymptomatik, sofern eine andere Ursache, die den Durchfall erklärt, nicht gefunden wird [34].

Beim nephrotischen Syndrom gab es, ebenfalls in diesen (italienischen) Leitlinien, Empfehlungen zur Kurzzeitinfusion von HA 20–25 % [34]. In den bereits erwähnten Querschnittleitlinien zur Therapie mit Blutkomponenten und Plasmaderivaten wird von einem Einsatz jedoch abgeraten, da zugeführtes Albumin wohl wieder renal ausgeschieden wird [20].

In den Querschnittleitlinien findet sich auch eine Empfehlung für HA als kolloidalen Volumenersatz zur Prävention und Therapie eines schweren ovariellen Hyperstimulationssyndroms, sollten andere Maßnahmen kontraindiziert sein. Es wird jedoch erneut die heterogene Datenlage bemerkt (Tab. 2, [20]).

**Table 2:** 

Krankheitsbild	Mögliche Einsatzgebiete
Sepsis	Falls Kristalloide zur Volumentherapie nicht ausreichen Nach Ausschöpfung anderer Maßnahmen
Leberzirrhose	Large-volume-Parazentese: bei punktiertem Aszitesvolumen > 5 l HRS-AKI in Kombination mit Terlipressin
Akute Nierenschädigung	HRS-AKI AKI in der Herzchirurgie bei Hypoalbuminämie (präliminäre Daten)
Nierenersatztherapie	Prävention von IDH bei hospitalisierten, hypoalbuminämischen Patienten (präliminäre Daten) Verbesserte negative Flüssigkeitsbilanzierung bei CRRT (präliminäre Daten)
Acute Respiratory Distress Syndrome (ARDS)	In Kombination mit Schleifendiuretika bei Hypoalbuminämie (präliminäre Daten)
Chirurgie	Perioperiativ erst nach Ausschöpfung anderer Maßnahmen
Herzchirurgie	Für hämodynamischen Stabilisierung und Priming der Herz-Lungen-Maschine (HA 5 %)
Verbrennungen	Nach Ausschöpfung anderer Maßnahmen Deresuscitation-Phase im Flüssigkeitsmanagement

*HA* Humanalbumin, *HRS-AKI* hepatorenales Syndrom – akute Nierenschädigung, *IDH* intradialytische Hypotonie, *CRRT* kontinuierliche Nierenersatztherapie

## Fazit für die Praxis

- Mit Ausnahme von hypoonkotischem Humanalbumin (HA) 4–5 % beim Schädel-Hirn-Trauma kann der Einsatz von Albumin als Volumentherapeutikum in fast allen Bereichen als sicher angesehen werden.
- Während die Indikationen vor allem für Personen mit Leberzirrhose klar sind, muss die Entscheidung für den Einsatz von HA in vielen anderen Einsatzbereichen auf individueller Basis getroffen werden.
- Zumeist kann der Einsatz von HA vor allem nach Ausschöpfung anderer Maßnahmen zur Volumentherapie und hämodynamischen Stabilisierung in Erwägung gezogen werden. Dies gilt derzeit sowohl für die Sepsis als auch für den peri- und postoperativen Einsatz und bei Verbrennungen.
- Inwiefern sich die Einsatzgebiete von HA in Zukunft erweitern werden, müssen große laufende und zukünftige Studien zeigen. Deren Finanzierung müsste wegen des generischen Status von HA wohl vermehrt von der öffentlichen Hand getragen werden.
- Bei den zukünftigen Studien dürfte die Hypoalbuminämie aufgrund der wachsenden Bedeutung bei individualisierten Therapieentscheidungen eine zunehmende Rolle spielen.
